# 3β-Acet­oxy-12α-chloro-d-friedooleanan-28,14β-olide

**DOI:** 10.1107/S160053681100585X

**Published:** 2011-02-23

**Authors:** Anna Froelich, Marcin Kowiel, Barbara Bednarczyk-Cwynar, Lucjusz Zaprutko, Andrzej K. Gzella

**Affiliations:** a Department of Organic Chemistry, Poznan University of Medical Sciences, ul. Grunwaldzka 6, 60-780 Poznań, Poland; b Faculty of Pharmacy, Ludwik Rydygier Collegium Medicum in Bydgoszcz, Nicolaus Copernicus University in Torun, ul. M. Curie Skłodowskiej 9, 85-094 Bydgoszcz, Poland

## Abstract

The title compound, C_32_H_49_ClO_4_, was obtained along with nitrile and lactam products in the POCl_3_-catalysed Beckmann rearrangement from 3β-acet­oxy-12-hydroxyiminoolean-28-olic acid methyl ester. The mechanism of the transformation leading to the title compound remains unclear and requires further investigation. Rings *A*, *B* and *E* are in chair conformations, ring *C* has a twisted-boat conformation, ring *D* a conformation halfway between boat and twisted-boat and rings *D* and *E* are *cis*-fused. In the crystal, mol­ecules are connected by weak inter­molecular C—H⋯O hydrogen bonds into layers extending parallel to the *bc* plane.

## Related literature

For background to the synthesis of lactam and thiol­actam derivatives of oleanolic acid, see: Bednarczyk-Cwynar (2007 ▶). For ring conformation analysis, see Cremer & Pople (1975 ▶).
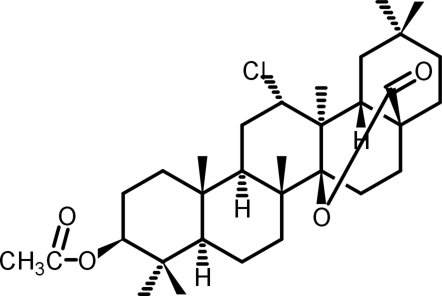


## Experimental

### 

#### Crystal data

C_32_H_49_ClO_4_
*M* *_r_* = 533.16
Monoclinic, 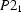

*a* = 14.1022 (2) Å
*b* = 6.6481 (1) Å
*c* = 15.2632 (2) Å
β = 90.621 (1)°
*V* = 1430.88 (4) Å^3^
*Z* = 2
Cu *K*α radiation
μ = 1.45 mm^−1^
*T* = 130 K
0.35 × 0.10 × 0.05 mm

#### Data collection

Oxford Diffraction SuperNova Single source at offset Atlas diffractometer
Absorption correction: multi-scan (*CrysAlis PRO*; Oxford Diffraction, 2007 ▶) *T*_min_ = 0.452, *T*_max_ = 1.000
10416 measured reflections
5530 independent reflections
5337 reflections with *I* > 2σ(*I*)
*R*_int_ = 0.032

#### Refinement

*R*[*F* ^2^ > 2σ(*F* ^2^)] = 0.043
*wR*(*F* ^2^) = 0.119
*S* = 1.04
5530 reflections
342 parameters
1 restraint
H-atom parameters constrained
Δρ_max_ = 0.41 e Å^−3^
Δρ_min_ = −0.35 e Å^−3^
Absolute structure: Flack (1983 ▶), 2413 Friedel pairs
Flack parameter: 0.024 (14)

### 

Data collection: *CrysAlis PRO* (Oxford Diffraction, 2007 ▶); cell refinement: *CrysAlis PRO*; data reduction: *CrysAlis PRO*; program(s) used to solve structure: *SHELXS97* (Sheldrick, 2008 ▶); program(s) used to refine structure: *SHELXL97* (Sheldrick, 2008 ▶); molecular graphics: *ORTEP-3 for Windows* (Farrugia, 1997 ▶); software used to prepare material for publication: *WinGX* (Farrugia, 1999 ▶) and *PLATON* (Spek, 2009 ▶).

## Supplementary Material

Crystal structure: contains datablocks I, global. DOI: 10.1107/S160053681100585X/bt5473sup1.cif

Structure factors: contains datablocks I. DOI: 10.1107/S160053681100585X/bt5473Isup2.hkl

Additional supplementary materials:  crystallographic information; 3D view; checkCIF report

## Figures and Tables

**Table 1:** Hydrogen-bond geometry (Å, °)

*D*—H⋯*A*	*D*—H	H⋯*A*	*D*⋯*A*	*D*—H⋯*A*
C16—H16*B*⋯O4^i^	0.97	2.41	3.358 (2)	167
C32—H32*A*⋯O4^ii^	0.96	2.55	3.390 (3)	146

Symmetry codes: (i) 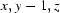; (ii) 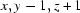.

**Fig. 1.**
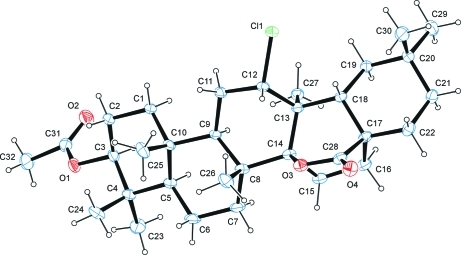
The molecular structure of (I), showing the atomic labelling scheme. Non-H atoms are drawn as 30% probability displacement ellipsoids and H atoms are drawn as spheres of an arbitrary size.

**Fig. 2.**
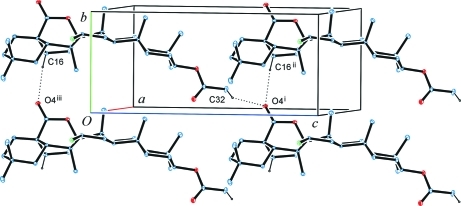
The hydrogen bonding (dotted lines) in the title structure. H atoms not involved in hydrogen bonds have been omitted for clarity. [Symmetry codes: (i) x, -1 + y, 1 + z; (ii) x, y, 1 + z]

## supplementary crystallographic information

### Comment

In a POCl_3_-catalysed Beckmann rearrangement of 3β-acetoxy-12-hydroxyiminoolean-28-olic acid methyl ester, an unexpected compound with a chlorine instead of nitrogen atom was obtained along with the typical nitrile and lactam products. The X-ray analysis allowed to identify the compound as 3β-acetoxy-12α-chloro-D-friedooleanan-28,14β-olide (3β-acetoxy-12α-chloro-14β-isooleanan-28,14β-olide) (I). The mechanism of the reaction that led to compound (I) requires further investigations.

The X-ray analysis has revealed that the title compound  contains a δ-lactone ring with the C28═O4 group adjacent to C17. Formation of the lactone bridge is possible because of the change in the configuration of the chiral centers C13 and C14. As a result, the ether oxygen atom O3 at C14 and the methyl group at C13 are axial with respect to rings *C* and *D*. The former substituent reveals β-configuration, while the latter one has α-configuration. This observation shows that methyl group C27 has undergone 1,2-shift from C14 to C13 retaining its original orientation.

The chlorine atom at C12, belonging to ring *C*, is oriented equatorially and assumes α-configuration.

Rings *A*, *B* and *E* of the triterpenoid skeleton adopt chair conformations, each with different degree of distortion. Ring *C* has a twisted-boat conformation. Puckering parameters (Cremer & Pople, 1975) are *Q* = 0.747 (2) Å, θ = 95.61 (15)°, φ = 36.80 (17)°, while ring *D* reveals a conformation halfway between boat and twisted-boat [Cremer & Pople puckering parameters: *Q* = 0.839 (2) Å, θ = 88.13 (14)°, φ = 48.76 (15)°].

The values of the dihedral angles in the title compound confirm the *trans* configuration of rings *A*/*B*, *B*/*C* and *C*/D [13.96 (8), 17.63 (4) and 13.26 (4)°] and the *cis* configuration of rings *D*/*E* [46.54 (6)°].

The acetoxy group at C3 is planar and adopts β-orientation. The carbonyl group C31═O2 of the above acetoxy group is synperiplanar with respect to the O1—C3 bond [torsion angle C3—O1—C31—O2: -5.0 (3)°] and adopts a conformation similar to synperiplanar with respect to the C2—C3 bond [torsion angle C2—C3—C31—O2: 72.9 (2)°].

In the crystal lattice, the molecules  are connected with three-centered weak hydrogen bonds C32—H32A···O4^i^···H16B^ii^—C16^ii^ [(i) *x*, -1+*y*, 1+*z*; (ii) *x*, *y*, 1+*z*] into layers extending parallel to the *bc* plane. The layer thickness is about a half of the *a* parameter length.

### Experimental

The title compound was obtained as a by-product in POCl_3_-catalysed Beckmann rearrangement reaction and recrystallized from ethanol solution at room temperature.

### Refinement

All H-atoms were placed in geometrically calculated positions and were refined with a riding model with C—H = 0.96–0.98 Å and with *U*_iso_ = 1.2 *U*_eq_(C) or 1.5 *U*_eq_(C) for methyl groups. The methyl H atoms were refined as rigid groups, which were allowed to rotate. The absolute configuration of the title compound was established by refinement of the Flack (1983) parameter.

### Crystal data

**Table:**

C_32_H_49_ClO_4_	*F*(000) = 580
*M**_r_* = 533.16	*D*_x_ = 1.237 Mg m^−^^3^
Monoclinic, *P*2_1_	Cu *K*α radiation, λ = 1.54184 Å
Hall symbol: P 2yb	Cell parameters from 7077 reflections
*a* = 14.1022 (2) Å	θ = 2.9–73.7°
*b* = 6.6481 (1) Å	µ = 1.45 mm^−^^1^
*c* = 15.2632 (2) Å	*T* = 130 K
β = 90.621 (1)°	Needle, colourless
*V* = 1430.88 (4) Å^3^	0.35 × 0.10 × 0.05 mm
*Z* = 2	

### Data collection

**Table:**

Oxford Diffraction SuperNova Single source at offset Atlas diffractometer	5530 independent reflections
Radiation source: SuperNova (Cu) X-ray Source	5337 reflections with *I* > 2σ(*I*)
mirror	*R*_int_ = 0.032
Detector resolution: 10.5357 pixels mm^-1^	θ_max_ = 73.8°, θ_min_ = 2.9°
ω scans	*h* = −17→17
Absorption correction: multi-scan (*CrysAlis PRO*; Oxford Diffraction, 2007)	*k* = −8→8
*T*_min_ = 0.452, *T*_max_ = 1.000	*l* = −18→18
10416 measured reflections	

### Refinement

**Table:**

Refinement on *F*^2^	Secondary atom site location: difference Fourier map
Least-squares matrix: full	Hydrogen site location: inferred from neighbouring sites
*R*[*F*^2^ > 2σ(*F*^2^)] = 0.043	H-atom parameters constrained
*wR*(*F*^2^) = 0.119	*w* = 1/[σ^2^(*F*_o_^2^) + (0.0742*P*)^2^ + 0.2024*P*] where *P* = (*F*_o_^2^ + 2*F*_c_^2^)/3
*S* = 1.04	(Δ/σ)_max_ < 0.001
5530 reflections	Δρ_max_ = 0.41 e Å^−^^3^
342 parameters	Δρ_min_ = −0.35 e Å^−^^3^
1 restraint	Absolute structure: Flack (1983), 2413 Friedel pairs
Primary atom site location: structure-invariant direct methods	Flack parameter: 0.024 (14)

### Special details

**Table:**

Experimental. CrysAlisPro (Oxford Diffraction, 2007) Empirical absorption correction using spherical harmonics, implemented in SCALE3 ABSPACK scaling algorithm.
Geometry. All e.s.d.'s (except the e.s.d. in the dihedral angle between two l.s. planes) are estimated using the full covariance matrix. The cell e.s.d.'s are taken into account individually in the estimation of e.s.d.'s in distances, angles and torsion angles; correlations between e.s.d.'s in cell parameters are only used when they are defined by crystal symmetry. An approximate (isotropic) treatment of cell e.s.d.'s is used for estimating e.s.d.'s involving l.s. planes.
Refinement. Refinement of *F*^2^ against ALL reflections. The weighted *R*-factor *wR* and goodness of fit *S* are based on *F*^2^, conventional *R*-factors *R* are based on *F*, with *F* set to zero for negative *F*^2^. The threshold expression of *F*^2^ > σ(*F*^2^) is used only for calculating *R*-factors(gt) *etc*. and is not relevant to the choice of reflections for refinement. *R*-factors based on *F*^2^ are statistically about twice as large as those based on *F*, and *R*- factors based on ALL data will be even larger.

### Fractional atomic coordinates and isotropic or equivalent isotropic displacement parameters (Å^2^)

**Table:**

	*x*	*y*	*z*	*U*_iso_*/*U*_eq_	
Cl1	−0.01593 (3)	0.72974 (8)	−0.07850 (3)	0.03101 (13)	
O1	0.34716 (12)	0.4238 (3)	0.40093 (10)	0.0353 (4)	
O2	0.26503 (15)	0.1327 (3)	0.40399 (12)	0.0463 (4)	
O3	0.27784 (11)	0.9153 (2)	−0.15431 (10)	0.0298 (3)	
O4	0.26583 (13)	1.0712 (2)	−0.28151 (11)	0.0372 (4)	
C1	0.19066 (14)	0.5857 (4)	0.21099 (14)	0.0296 (4)	
H1A	0.1293	0.6516	0.2094	0.036*	
H1B	0.1835	0.4547	0.1837	0.036*	
C2	0.22185 (15)	0.5574 (4)	0.30664 (14)	0.0322 (5)	
H2A	0.2251	0.6871	0.3357	0.039*	
H2B	0.1760	0.4749	0.3370	0.039*	
C3	0.31775 (16)	0.4575 (3)	0.30991 (14)	0.0302 (4)	
H3	0.3122	0.3263	0.2810	0.036*	
C4	0.39682 (15)	0.5760 (4)	0.26497 (15)	0.0330 (5)	
C5	0.36160 (14)	0.6135 (3)	0.16899 (14)	0.0284 (4)	
H5	0.3543	0.4785	0.1440	0.034*	
C6	0.43350 (14)	0.7172 (5)	0.10948 (16)	0.0399 (5)	
H6A	0.4371	0.8589	0.1243	0.048*	
H6B	0.4957	0.6585	0.1191	0.048*	
C7	0.40540 (14)	0.6949 (4)	0.01315 (16)	0.0382 (6)	
H7A	0.4075	0.5535	−0.0024	0.046*	
H7B	0.4517	0.7645	−0.0223	0.046*	
C8	0.30584 (14)	0.7777 (3)	−0.00945 (15)	0.0290 (4)	
C9	0.23589 (13)	0.6866 (3)	0.05772 (13)	0.0224 (4)	
H9	0.2401	0.5413	0.0478	0.027*	
C10	0.26190 (13)	0.7119 (3)	0.15757 (13)	0.0256 (4)	
C11	0.13101 (13)	0.7367 (4)	0.03798 (13)	0.0270 (4)	
H11A	0.0917	0.6278	0.0592	0.032*	
H11B	0.1139	0.8575	0.0697	0.032*	
C12	0.11030 (13)	0.7687 (3)	−0.05947 (13)	0.0241 (4)	
H12	0.1244	0.9096	−0.0730	0.029*	
C13	0.17042 (14)	0.6377 (3)	−0.11985 (14)	0.0261 (4)	
C14	0.27356 (13)	0.7205 (3)	−0.10760 (13)	0.0277 (4)	
C15	0.34176 (15)	0.5844 (3)	−0.15818 (15)	0.0304 (4)	
H15A	0.3441	0.4524	−0.1311	0.037*	
H15B	0.4051	0.6412	−0.1564	0.037*	
C16	0.30721 (16)	0.5649 (3)	−0.25483 (16)	0.0323 (5)	
H16A	0.3600	0.5894	−0.2936	0.039*	
H16B	0.2848	0.4289	−0.2652	0.039*	
C17	0.22661 (14)	0.7154 (3)	−0.27601 (14)	0.0281 (4)	
C18	0.13854 (14)	0.6671 (3)	−0.21902 (13)	0.0237 (4)	
H18	0.0968	0.7849	−0.2214	0.028*	
C19	0.08285 (15)	0.4893 (3)	−0.25754 (14)	0.0285 (4)	
H19A	0.0270	0.4666	−0.2224	0.034*	
H19B	0.1219	0.3694	−0.2540	0.034*	
C20	0.05171 (17)	0.5218 (4)	−0.35383 (15)	0.0364 (5)	
C21	0.14176 (17)	0.5539 (4)	−0.40761 (14)	0.0339 (5)	
H21A	0.1240	0.5786	−0.4682	0.041*	
H21B	0.1796	0.4322	−0.4057	0.041*	
C22	0.20191 (16)	0.7308 (4)	−0.37383 (14)	0.0344 (4)	
H22A	0.2602	0.7367	−0.4069	0.041*	
H22B	0.1676	0.8551	−0.3842	0.041*	
C23	0.48479 (19)	0.4402 (5)	0.26288 (18)	0.0484 (7)	
H23A	0.4724	0.3265	0.2257	0.073*	
H23B	0.4994	0.3943	0.3211	0.073*	
H23C	0.5376	0.5148	0.2405	0.073*	
C24	0.42270 (18)	0.7675 (4)	0.31517 (18)	0.0431 (6)	
H24A	0.4655	0.8468	0.2810	0.065*	
H24B	0.4526	0.7323	0.3698	0.065*	
H24C	0.3662	0.8436	0.3262	0.065*	
C25	0.25426 (19)	0.9299 (4)	0.19146 (16)	0.0384 (5)	
H25A	0.3139	0.9971	0.1841	0.058*	
H25B	0.2383	0.9285	0.2524	0.058*	
H25C	0.2058	0.9998	0.1589	0.058*	
C26	0.3105 (2)	1.0101 (4)	0.00024 (18)	0.0398 (5)	
H26A	0.2478	1.0653	−0.0060	0.060*	
H26B	0.3505	1.0648	−0.0443	0.060*	
H26C	0.3359	1.0439	0.0570	0.060*	
C27	0.16265 (15)	0.4122 (3)	−0.09463 (13)	0.0279 (4)	
H27A	0.1900	0.3917	−0.0375	0.042*	
H27B	0.1960	0.3321	−0.1366	0.042*	
H27C	0.0971	0.3731	−0.0942	0.042*	
C28	0.25886 (16)	0.9156 (3)	−0.24151 (15)	0.0302 (4)	
C29	0.0028 (2)	0.3326 (5)	−0.38573 (18)	0.0538 (8)	
H29A	0.0450	0.2201	−0.3790	0.081*	
H29B	−0.0144	0.3476	−0.4464	0.081*	
H29C	−0.0533	0.3098	−0.3520	0.081*	
C30	−0.01811 (19)	0.6979 (5)	−0.36211 (16)	0.0477 (7)	
H30A	−0.0713	0.6747	−0.3247	0.072*	
H30B	−0.0398	0.7088	−0.4218	0.072*	
H30C	0.0131	0.8204	−0.3450	0.072*	
C31	0.31790 (16)	0.2516 (4)	0.43831 (14)	0.0357 (5)	
C32	0.36249 (19)	0.2262 (6)	0.52764 (16)	0.0495 (6)	
H32A	0.3218	0.1459	0.5636	0.074*	
H32B	0.3713	0.3558	0.5542	0.074*	
H32C	0.4228	0.1606	0.5221	0.074*	

### Atomic displacement parameters (Å^2^)

**Table:**

	*U*^11^	*U*^22^	*U*^33^	*U*^12^	*U*^13^	*U*^23^
Cl1	0.0209 (2)	0.0390 (3)	0.0330 (2)	0.0011 (2)	−0.00120 (16)	−0.0023 (2)
O1	0.0357 (8)	0.0442 (9)	0.0259 (7)	0.0047 (7)	−0.0085 (6)	−0.0020 (7)
O2	0.0516 (10)	0.0507 (11)	0.0365 (9)	−0.0055 (9)	−0.0073 (8)	0.0023 (8)
O3	0.0297 (8)	0.0218 (7)	0.0381 (8)	−0.0039 (6)	0.0031 (6)	0.0019 (6)
O4	0.0483 (10)	0.0235 (7)	0.0398 (8)	−0.0068 (7)	0.0048 (7)	0.0053 (6)
C1	0.0181 (9)	0.0431 (11)	0.0275 (10)	0.0026 (8)	−0.0025 (7)	−0.0031 (9)
C2	0.0220 (9)	0.0473 (13)	0.0271 (10)	0.0022 (9)	−0.0037 (8)	−0.0037 (9)
C3	0.0300 (10)	0.0364 (11)	0.0241 (9)	0.0037 (9)	−0.0059 (8)	−0.0034 (8)
C4	0.0203 (9)	0.0431 (12)	0.0355 (11)	0.0055 (9)	−0.0052 (8)	−0.0014 (10)
C5	0.0191 (9)	0.0333 (11)	0.0328 (10)	0.0023 (8)	−0.0021 (7)	0.0002 (8)
C6	0.0167 (8)	0.0579 (15)	0.0451 (12)	−0.0006 (11)	−0.0044 (8)	0.0114 (12)
C7	0.0174 (9)	0.0555 (15)	0.0418 (12)	−0.0025 (10)	0.0030 (8)	0.0086 (10)
C8	0.0193 (9)	0.0303 (11)	0.0373 (11)	−0.0012 (7)	−0.0015 (8)	0.0074 (8)
C9	0.0170 (8)	0.0216 (9)	0.0286 (9)	0.0007 (6)	−0.0007 (7)	−0.0021 (7)
C10	0.0192 (8)	0.0270 (10)	0.0305 (9)	0.0025 (8)	−0.0050 (7)	−0.0052 (8)
C11	0.0203 (8)	0.0306 (9)	0.0300 (9)	0.0040 (9)	−0.0023 (7)	−0.0041 (9)
C12	0.0180 (8)	0.0238 (10)	0.0305 (9)	0.0021 (7)	−0.0011 (7)	−0.0001 (7)
C13	0.0209 (9)	0.0279 (9)	0.0294 (10)	−0.0014 (8)	0.0019 (7)	−0.0018 (8)
C14	0.0211 (8)	0.0288 (10)	0.0333 (10)	−0.0001 (9)	0.0022 (7)	0.0047 (9)
C15	0.0212 (9)	0.0272 (9)	0.0429 (11)	0.0009 (8)	0.0047 (8)	−0.0003 (9)
C16	0.0275 (11)	0.0285 (10)	0.0412 (11)	−0.0015 (8)	0.0091 (9)	−0.0047 (9)
C17	0.0276 (9)	0.0221 (9)	0.0348 (10)	−0.0039 (8)	0.0087 (8)	0.0009 (8)
C18	0.0221 (9)	0.0212 (8)	0.0278 (9)	−0.0014 (7)	0.0037 (7)	0.0015 (7)
C19	0.0276 (10)	0.0305 (10)	0.0274 (10)	−0.0075 (8)	0.0022 (8)	−0.0007 (8)
C20	0.0303 (11)	0.0519 (14)	0.0270 (10)	−0.0083 (10)	0.0001 (9)	−0.0029 (9)
C21	0.0364 (12)	0.0390 (12)	0.0263 (10)	0.0000 (10)	0.0033 (8)	0.0013 (9)
C22	0.0408 (11)	0.0295 (10)	0.0330 (10)	−0.0025 (10)	0.0101 (8)	0.0009 (9)
C23	0.0321 (12)	0.0718 (19)	0.0413 (13)	0.0215 (13)	−0.0058 (10)	0.0050 (13)
C24	0.0338 (11)	0.0483 (15)	0.0469 (13)	−0.0085 (11)	−0.0169 (10)	−0.0006 (11)
C25	0.0430 (13)	0.0325 (11)	0.0394 (12)	0.0080 (10)	−0.0144 (10)	−0.0102 (10)
C26	0.0450 (13)	0.0319 (11)	0.0422 (13)	−0.0106 (10)	−0.0083 (11)	0.0030 (10)
C27	0.0281 (10)	0.0255 (9)	0.0302 (10)	−0.0036 (8)	0.0023 (8)	−0.0004 (8)
C28	0.0280 (10)	0.0254 (9)	0.0372 (11)	−0.0033 (8)	0.0089 (8)	0.0014 (9)
C29	0.0549 (18)	0.0704 (19)	0.0361 (13)	−0.0270 (15)	0.0009 (12)	−0.0084 (13)
C30	0.0374 (12)	0.072 (2)	0.0342 (11)	0.0081 (13)	−0.0040 (9)	0.0052 (12)
C31	0.0312 (10)	0.0468 (13)	0.0290 (10)	0.0090 (11)	−0.0011 (8)	−0.0004 (10)
C32	0.0479 (13)	0.0700 (18)	0.0304 (11)	0.0069 (16)	−0.0079 (10)	0.0091 (14)

### Geometric parameters (Å, °)

**Table:**

Cl1—C12	1.8191 (19)	C15—H15B	0.9700
O1—C31	1.346 (3)	C16—C17	1.546 (3)
O1—C3	1.463 (3)	C16—H16A	0.9700
O2—C31	1.203 (3)	C16—H16B	0.9700
O3—C28	1.355 (3)	C17—C28	1.500 (3)
O3—C14	1.480 (3)	C17—C22	1.533 (3)
O4—C28	1.206 (3)	C17—C18	1.557 (2)
C1—C2	1.532 (3)	C18—C19	1.533 (3)
C1—C10	1.548 (3)	C18—H18	0.9800
C1—H1A	0.9700	C19—C20	1.545 (3)
C1—H1B	0.9700	C19—H19A	0.9700
C2—C3	1.507 (3)	C19—H19B	0.9700
C2—H2A	0.9700	C20—C29	1.513 (4)
C2—H2B	0.9700	C20—C30	1.534 (4)
C3—C4	1.533 (3)	C20—C21	1.534 (3)
C3—H3	0.9800	C21—C22	1.536 (3)
C4—C24	1.528 (4)	C21—H21A	0.9700
C4—C23	1.535 (3)	C21—H21B	0.9700
C4—C5	1.562 (3)	C22—H22A	0.9700
C5—C6	1.532 (3)	C22—H22B	0.9700
C5—C10	1.559 (3)	C23—H23A	0.9600
C5—H5	0.9800	C23—H23B	0.9600
C6—C7	1.526 (3)	C23—H23C	0.9600
C6—H6A	0.9700	C24—H24A	0.9600
C6—H6B	0.9700	C24—H24B	0.9600
C7—C8	1.544 (3)	C24—H24C	0.9600
C7—H7A	0.9700	C25—H25A	0.9600
C7—H7B	0.9700	C25—H25B	0.9600
C8—C9	1.553 (3)	C25—H25C	0.9600
C8—C26	1.554 (3)	C26—H26A	0.9600
C8—C14	1.607 (3)	C26—H26B	0.9600
C9—C11	1.542 (2)	C26—H26C	0.9600
C9—C10	1.573 (3)	C27—H27A	0.9600
C9—H9	0.9800	C27—H27B	0.9600
C10—C25	1.543 (3)	C27—H27C	0.9600
C11—C12	1.528 (3)	C29—H29A	0.9600
C11—H11A	0.9700	C29—H29B	0.9600
C11—H11B	0.9700	C29—H29C	0.9600
C12—C13	1.531 (3)	C30—H30A	0.9600
C12—H12	0.9800	C30—H30B	0.9600
C13—C27	1.552 (3)	C30—H30C	0.9600
C13—C14	1.564 (3)	C31—C32	1.505 (3)
C13—C18	1.587 (3)	C32—H32A	0.9600
C14—C15	1.535 (3)	C32—H32B	0.9600
C15—C16	1.554 (3)	C32—H32C	0.9600
C15—H15A	0.9700		
			
C31—O1—C3	116.63 (18)	C15—C16—H16A	109.3
C28—O3—C14	117.76 (16)	C17—C16—H16B	109.3
C2—C1—C10	112.78 (17)	C15—C16—H16B	109.3
C2—C1—H1A	109.0	H16A—C16—H16B	108.0
C10—C1—H1A	109.0	C28—C17—C22	110.32 (19)
C2—C1—H1B	109.0	C28—C17—C16	106.33 (18)
C10—C1—H1B	109.0	C22—C17—C16	113.95 (18)
H1A—C1—H1B	107.8	C28—C17—C18	103.14 (16)
C3—C2—C1	109.51 (17)	C22—C17—C18	112.56 (16)
C3—C2—H2A	109.8	C16—C17—C18	109.82 (17)
C1—C2—H2A	109.8	C19—C18—C17	110.67 (17)
C3—C2—H2B	109.8	C19—C18—C13	114.17 (16)
C1—C2—H2B	109.8	C17—C18—C13	109.81 (16)
H2A—C2—H2B	108.2	C19—C18—H18	107.3
O1—C3—C2	110.13 (17)	C17—C18—H18	107.3
O1—C3—C4	107.68 (17)	C13—C18—H18	107.3
C2—C3—C4	114.56 (19)	C18—C19—C20	113.35 (18)
O1—C3—H3	108.1	C18—C19—H19A	108.9
C2—C3—H3	108.1	C20—C19—H19A	108.9
C4—C3—H3	108.1	C18—C19—H19B	108.9
C24—C4—C3	112.0 (2)	C20—C19—H19B	108.9
C24—C4—C23	108.2 (2)	H19A—C19—H19B	107.7
C3—C4—C23	107.4 (2)	C29—C20—C30	108.6 (2)
C24—C4—C5	114.1 (2)	C29—C20—C21	108.7 (2)
C3—C4—C5	106.04 (17)	C30—C20—C21	112.6 (2)
C23—C4—C5	108.86 (19)	C29—C20—C19	108.3 (2)
C6—C5—C10	110.29 (18)	C30—C20—C19	111.1 (2)
C6—C5—C4	114.97 (17)	C21—C20—C19	107.46 (18)
C10—C5—C4	116.68 (17)	C20—C21—C22	112.59 (19)
C6—C5—H5	104.4	C20—C21—H21A	109.1
C10—C5—H5	104.4	C22—C21—H21A	109.1
C4—C5—H5	104.4	C20—C21—H21B	109.1
C7—C6—C5	111.14 (19)	C22—C21—H21B	109.1
C7—C6—H6A	109.4	H21A—C21—H21B	107.8
C5—C6—H6A	109.4	C17—C22—C21	113.23 (19)
C7—C6—H6B	109.4	C17—C22—H22A	108.9
C5—C6—H6B	109.4	C21—C22—H22A	108.9
H6A—C6—H6B	108.0	C17—C22—H22B	108.9
C6—C7—C8	114.00 (19)	C21—C22—H22B	108.9
C6—C7—H7A	108.8	H22A—C22—H22B	107.7
C8—C7—H7A	108.8	C4—C23—H23A	109.5
C6—C7—H7B	108.8	C4—C23—H23B	109.5
C8—C7—H7B	108.8	H23A—C23—H23B	109.5
H7A—C7—H7B	107.6	C4—C23—H23C	109.5
C7—C8—C9	107.22 (17)	H23A—C23—H23C	109.5
C7—C8—C26	107.2 (2)	H23B—C23—H23C	109.5
C9—C8—C26	110.55 (19)	C4—C24—H24A	109.5
C7—C8—C14	111.84 (18)	C4—C24—H24B	109.5
C9—C8—C14	110.34 (16)	H24A—C24—H24B	109.5
C26—C8—C14	109.59 (18)	C4—C24—H24C	109.5
C11—C9—C8	113.67 (17)	H24A—C24—H24C	109.5
C11—C9—C10	112.29 (15)	H24B—C24—H24C	109.5
C8—C9—C10	117.06 (16)	C10—C25—H25A	109.5
C11—C9—H9	104.0	C10—C25—H25B	109.5
C8—C9—H9	104.0	H25A—C25—H25B	109.5
C10—C9—H9	104.0	C10—C25—H25C	109.5
C25—C10—C1	106.53 (18)	H25A—C25—H25C	109.5
C25—C10—C5	115.01 (17)	H25B—C25—H25C	109.5
C1—C10—C5	107.68 (17)	C8—C26—H26A	109.5
C25—C10—C9	114.12 (17)	C8—C26—H26B	109.5
C1—C10—C9	107.86 (15)	H26A—C26—H26B	109.5
C5—C10—C9	105.32 (15)	C8—C26—H26C	109.5
C12—C11—C9	113.15 (15)	H26A—C26—H26C	109.5
C12—C11—H11A	108.9	H26B—C26—H26C	109.5
C9—C11—H11A	108.9	C13—C27—H27A	109.5
C12—C11—H11B	108.9	C13—C27—H27B	109.5
C9—C11—H11B	108.9	H27A—C27—H27B	109.5
H11A—C11—H11B	107.8	C13—C27—H27C	109.5
C11—C12—C13	113.91 (16)	H27A—C27—H27C	109.5
C11—C12—Cl1	108.18 (13)	H27B—C27—H27C	109.5
C13—C12—Cl1	111.72 (14)	O4—C28—O3	118.8 (2)
C11—C12—H12	107.6	O4—C28—C17	127.6 (2)
C13—C12—H12	107.6	O3—C28—C17	113.55 (18)
Cl1—C12—H12	107.6	C20—C29—H29A	109.5
C12—C13—C27	111.07 (17)	C20—C29—H29B	109.5
C12—C13—C14	104.35 (16)	H29A—C29—H29B	109.5
C27—C13—C14	112.24 (17)	C20—C29—H29C	109.5
C12—C13—C18	110.54 (16)	H29A—C29—H29C	109.5
C27—C13—C18	109.62 (16)	H29B—C29—H29C	109.5
C14—C13—C18	108.90 (15)	C20—C30—H30A	109.5
O3—C14—C15	104.16 (15)	C20—C30—H30B	109.5
O3—C14—C13	107.05 (16)	H30A—C30—H30B	109.5
C15—C14—C13	108.61 (18)	C20—C30—H30C	109.5
O3—C14—C8	103.27 (17)	H30A—C30—H30C	109.5
C15—C14—C8	115.83 (17)	H30B—C30—H30C	109.5
C13—C14—C8	116.64 (15)	O2—C31—O1	124.5 (2)
C14—C15—C16	109.57 (17)	O2—C31—C32	124.9 (3)
C14—C15—H15A	109.8	O1—C31—C32	110.7 (2)
C16—C15—H15A	109.8	C31—C32—H32A	109.5
C14—C15—H15B	109.8	C31—C32—H32B	109.5
C16—C15—H15B	109.8	H32A—C32—H32B	109.5
H15A—C15—H15B	108.2	C31—C32—H32C	109.5
C17—C16—C15	111.53 (17)	H32A—C32—H32C	109.5
C17—C16—H16A	109.3	H32B—C32—H32C	109.5
			
C10—C1—C2—C3	−58.0 (3)	C27—C13—C14—O3	165.62 (16)
C31—O1—C3—C2	87.0 (2)	C18—C13—C14—O3	44.0 (2)
C31—O1—C3—C4	−147.49 (19)	C12—C13—C14—C15	174.07 (17)
C1—C2—C3—O1	−178.22 (19)	C27—C13—C14—C15	53.7 (2)
C1—C2—C3—C4	60.2 (2)	C18—C13—C14—C15	−67.9 (2)
O1—C3—C4—C24	−53.5 (2)	C12—C13—C14—C8	41.0 (2)
C2—C3—C4—C24	69.4 (2)	C27—C13—C14—C8	−79.4 (2)
O1—C3—C4—C23	65.1 (2)	C18—C13—C14—C8	159.04 (17)
C2—C3—C4—C23	−172.0 (2)	C7—C8—C14—O3	−108.90 (18)
O1—C3—C4—C5	−178.59 (17)	C9—C8—C14—O3	131.84 (15)
C2—C3—C4—C5	−55.7 (2)	C26—C8—C14—O3	9.9 (2)
C24—C4—C5—C6	60.6 (3)	C7—C8—C14—C15	4.3 (3)
C3—C4—C5—C6	−175.7 (2)	C9—C8—C14—C15	−115.00 (19)
C23—C4—C5—C6	−60.4 (3)	C26—C8—C14—C15	123.0 (2)
C24—C4—C5—C10	−70.9 (3)	C7—C8—C14—C13	134.01 (19)
C3—C4—C5—C10	52.9 (2)	C9—C8—C14—C13	14.8 (2)
C23—C4—C5—C10	168.2 (2)	C26—C8—C14—C13	−107.2 (2)
C10—C5—C6—C7	−61.6 (3)	O3—C14—C15—C16	−60.3 (2)
C4—C5—C6—C7	164.0 (2)	C13—C14—C15—C16	53.5 (2)
C5—C6—C7—C8	57.3 (3)	C8—C14—C15—C16	−172.97 (17)
C6—C7—C8—C9	−50.2 (3)	C14—C15—C16—C17	10.2 (2)
C6—C7—C8—C26	68.5 (3)	C15—C16—C17—C28	47.7 (2)
C6—C7—C8—C14	−171.3 (2)	C15—C16—C17—C22	169.42 (18)
C7—C8—C9—C11	−173.74 (19)	C15—C16—C17—C18	−63.3 (2)
C26—C8—C9—C11	69.7 (2)	C28—C17—C18—C19	167.84 (17)
C14—C8—C9—C11	−51.7 (2)	C22—C17—C18—C19	49.0 (2)
C7—C8—C9—C10	52.7 (2)	C16—C17—C18—C19	−79.1 (2)
C26—C8—C9—C10	−63.9 (2)	C28—C17—C18—C13	−65.2 (2)
C14—C8—C9—C10	174.74 (16)	C22—C17—C18—C13	175.90 (18)
C2—C1—C10—C25	−70.6 (2)	C16—C17—C18—C13	47.8 (2)
C2—C1—C10—C5	53.3 (2)	C12—C13—C18—C19	−106.4 (2)
C2—C1—C10—C9	166.51 (17)	C27—C13—C18—C19	16.4 (2)
C6—C5—C10—C25	−67.8 (3)	C14—C13—C18—C19	139.51 (18)
C4—C5—C10—C25	65.8 (3)	C12—C13—C18—C17	128.64 (17)
C6—C5—C10—C1	173.67 (18)	C27—C13—C18—C17	−108.59 (18)
C4—C5—C10—C1	−52.7 (2)	C14—C13—C18—C17	14.6 (2)
C6—C5—C10—C9	58.8 (2)	C17—C18—C19—C20	−56.0 (2)
C4—C5—C10—C9	−167.64 (18)	C13—C18—C19—C20	179.47 (17)
C11—C9—C10—C25	−64.6 (2)	C18—C19—C20—C29	176.8 (2)
C8—C9—C10—C25	69.6 (2)	C18—C19—C20—C30	−64.1 (3)
C11—C9—C10—C1	53.6 (2)	C18—C19—C20—C21	59.5 (3)
C8—C9—C10—C1	−172.27 (17)	C29—C20—C21—C22	−174.1 (2)
C11—C9—C10—C5	168.34 (17)	C30—C20—C21—C22	65.6 (3)
C8—C9—C10—C5	−57.5 (2)	C19—C20—C21—C22	−57.1 (3)
C8—C9—C11—C12	28.7 (3)	C28—C17—C22—C21	−163.09 (19)
C10—C9—C11—C12	164.46 (17)	C16—C17—C22—C21	77.4 (2)
C9—C11—C12—C13	33.5 (3)	C18—C17—C22—C21	−48.5 (3)
C9—C11—C12—Cl1	158.41 (15)	C20—C21—C22—C17	53.8 (3)
C11—C12—C13—C27	53.4 (2)	C14—O3—C28—O4	−176.93 (19)
Cl1—C12—C13—C27	−69.55 (19)	C14—O3—C28—C17	5.6 (3)
C11—C12—C13—C14	−67.8 (2)	C22—C17—C28—O4	0.0 (3)
Cl1—C12—C13—C14	169.29 (13)	C16—C17—C28—O4	124.0 (2)
C11—C12—C13—C18	175.31 (16)	C18—C17—C28—O4	−120.4 (2)
Cl1—C12—C13—C18	52.36 (19)	C22—C17—C28—O3	177.17 (17)
C28—O3—C14—C15	55.2 (2)	C16—C17—C28—O3	−58.8 (2)
C28—O3—C14—C13	−59.7 (2)	C18—C17—C28—O3	56.7 (2)
C28—O3—C14—C8	176.65 (16)	C3—O1—C31—O2	−5.0 (3)
C12—C13—C14—O3	−74.01 (19)	C3—O1—C31—C32	173.44 (19)

### Hydrogen-bond geometry (Å, °)

**Table:**

*D*—H···*A*	*D*—H	H···*A*	*D*···*A*	*D*—H···*A*
C16—H16B···O4^i^	0.97	2.41	3.358 (2)	167
C32—H32A···O4^ii^	0.96	2.55	3.390 (3)	146

Symmetry codes: (i) *x*, *y*−1, *z*; (ii) *x*, *y*−1, *z*+1.
